# An Unexpected Migration of a Fully Covered Self-Expandable Metal Stent (FCSEMS) Placed for Esophageal Perforation: A Case Report

**DOI:** 10.7759/cureus.117488

**Published:** 2026-10-05

**Authors:** Antonis Neokleous, Georgios Penesis, Anna Pagoni, Michael Christoulakes, Maria Tzouvala

**Affiliations:** 1 Gastroenterology and Hepatology, General Hospital of Nikaia Agios Panteleimon, Athens, GRC

**Keywords:** airway obstruction, endoscopic management, esophageal perforation, over-the-scope clips, self-expandable metal stent, stent migration

## Abstract

Esophageal fully covered self-expandable metal stents (FCSEMSs) are considered effective in managing perforations. Stent migration distally is a common adverse event of FCSEMSs and occurs more frequently compared to partially covered stents. Techniques, such as endoscopic suturing and over-the-scope clips (OTSCs), are used to improve retention. We report a rare case of proximal migration of FCSEMS into the pharynx that occurred unexpectedly after FCSEMS placement to seal an esophageal perforation.

## Introduction

Perforation of the esophagus in adults is most frequently caused by ingested foreign bodies. These bodies have the ability to migrate through the esophageal wall, damaging the adjacent structures, including the aorta and the trachea, and occasionally causing mediastinitis with fatal outcomes [1].

The European Society of Gastrointestinal Endoscopy (ESGE) strongly recommends the placement of esophageal stents for perforations, as well as for malignant dysphagia, tracheoesophageal and broncho-esophageal fistulas, benign strictures, leaks, and variceal bleeding [2]. Through the years, stents have evolved from exclusively plastic material to flexible metallic stents, available as fully covered, partially covered, and uncovered types. Fully covered self-expandable metal stents (FCSEMSs) are mainly indicated for the management of benign conditions as they can be easily removed after the indicated period of time. Partially covered and uncovered stents allow the ingrowth and overgrowth of tissue, restricting their removal so they are mostly permanently placed.

Although stents are highly efficacious and applicable in a variety of conditions, the risk of distal migration restricts their clinical utility. The reported migration rates vary in the current literature, between 20% and 60%, depending on the indication and stent type [3,4]. Recent evidence also suggests that late migration, defined as occurring >4 weeks, may result in severe and potentially life-threatening complications, including gastrointestinal obstruction, perforation, respiratory compromise, and major hemorrhage [5]. Various techniques to overcome this limitation, including the use of flared ends or wire hooks, have been developed by the manufacturers. In addition, endoscopic techniques, namely endoscopic suturing, over-the-scope clips, and conventional through-the-scope clips, are associated with lower migration rates [6]. Unlike distal migration, proximal (oral-side) migration may result in displacement of the stent toward the hypopharynx and laryngeal inlet, where it may mechanically compromise the upper airway.

We report a rare case of proximal and not distal esophageal stent migration into the hypopharynx, causing proximal upper airway obstruction despite having a securely attached FCSEMS with clips.

## Case presentation

A 64-year-old male submitted to an elective lumbar surgical procedure at the Neurosurgery Department of our hospital presented with dysphagia and mild thoracic pain 48 hours after ingestion of analgesic tablets. An unrelated medical history of arterial hypertension, dyslipidemia, diabetes mellitus type II, and smoking was reported. The physical examination and ECG were unremarkable, whereas a high level of C-reactive protein (CRP) and leukocytosis (high level of white blood cell count (WBCs)) were observed (Table 1).

**Table 1:** Laboratory findings. Values are reported as recorded in the laboratory report. Absolute leukocyte differential counts are shown in parentheses.

Parameter	Result	Unit
White blood cells (WBC)	20.32	K/µL
Neutrophils	79.0 (16.10)	% (K/µL)
Lymphocytes	12.8 (2.61)	% (K/µL)
Hemoglobin	11.4	g/dL
Hematocrit	32.0	%
Platelets	347	K/µL
Glucose	173	mg/dL
Urea	38	mg/dL
Creatinine	1.2	mg/dL
Sodium (Na)	135	mmol/L
Potassium (K)	3.7	mmol/L
Creatine kinase (CK)	113	U/L
Lactate dehydrogenase (LDH)	237	U/L
AST (SGOT)	24	U/L
ALT (SGPT)	35	U/L
Alkaline phosphatase	75	U/L
γ-Glutamyl transferase (γ-GT)	90	U/L
Total bilirubin	0.6	mg/dL
Total serum protein	6.4	g/dL
Serum albumin	4.1	g/dL
Calcium	9.3	mg/dL
Magnesium	1.9	mg/dL
C-reactive protein (CRP)	126.0	mg/L

A CT scan demonstrated two intraluminal spherical structures - foreign bodies - in the distal third of the esophagus, accompanied by extraluminal air bubbles in the mediastinum, thereby confirming esophageal perforation. Endoscopy revealed two tablets of paracetamol still in their blister packaging, stuck with both edges perforating the esophageal wall (Figure 1). With the assistance of an overtube, the foreign object was successfully removed, revealing a disruption of tissue continuity and evidence of two perforation sites in the distal third of the esophagus. An FCSEMS (Boston Scientific WallFlex Fully Covered Esophageal Stent, 23 mm x 105 mm)was chosen and subsequently deployed under radiological guidance. The correct position of the stent was verified with postoperative radiography. At the time of the procedure, due to the unavailability of an over-the-scope clipping device (stentfix OTSC® System, OVESCO) which can provide greater compression and circumferential grip on the esophageal wall, offering more robust fixation, two conventional through-the-scope clips (TTSCs) were applied to grasp the proximal edge of the stent together with the adjacent esophageal mucosa, thereby securing the proximal end of the stent to the esophageal wall. The patient was thereafter extubated uneventfully and returned to the ward. Later that evening, he acutely developed hoarseness of breath, sialorrhea and shortness of breath. Oxygen therapy was initiated via a non-rebreather mask and an urgent chest CT scan was performed, revealing the migration of the esophageal stent in the upper esophagus and hypopharynx (Figure 2). Pharyngoscopy, using a video laryngoscope and foreign-body forceps, allowed retrieval of the stent and immediate relief of patient’s respiratory distress. It is believed that proximal migration of the stent occurred following an episode of vomiting. In the subsequent period, after a multidisciplinary reassessment and the patient’s consent, a decision with the treating physicians was made, to reattempt esophageal stent placement. A repeat esophageal stent placement with the use of an over-the-scope clip for fixation, was then performed to ensure continued coverage of the perforation. The patient's subsequent clinical course was uneventful, with progressive clinical improvement and no further episodes of stent migration or respiratory compromise. Follow-up assessment demonstrated successful healing of the esophageal perforation, and the stent was subsequently removed without complications after six weeks.

**Figure 1:**
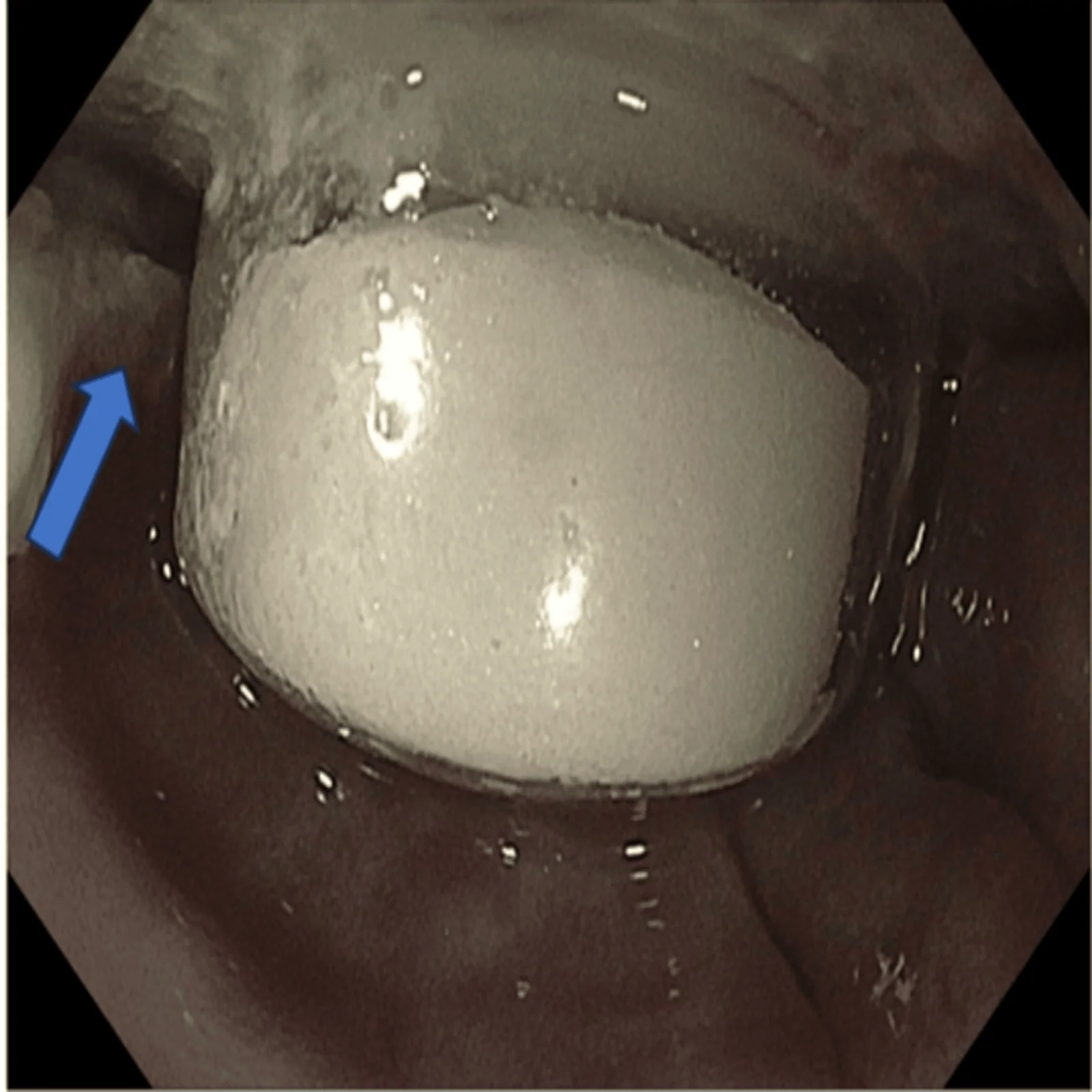
Endoscopic view demonstrating the foreign body penetrating the esophageal wall (arrowhead).

**Figure 2:**
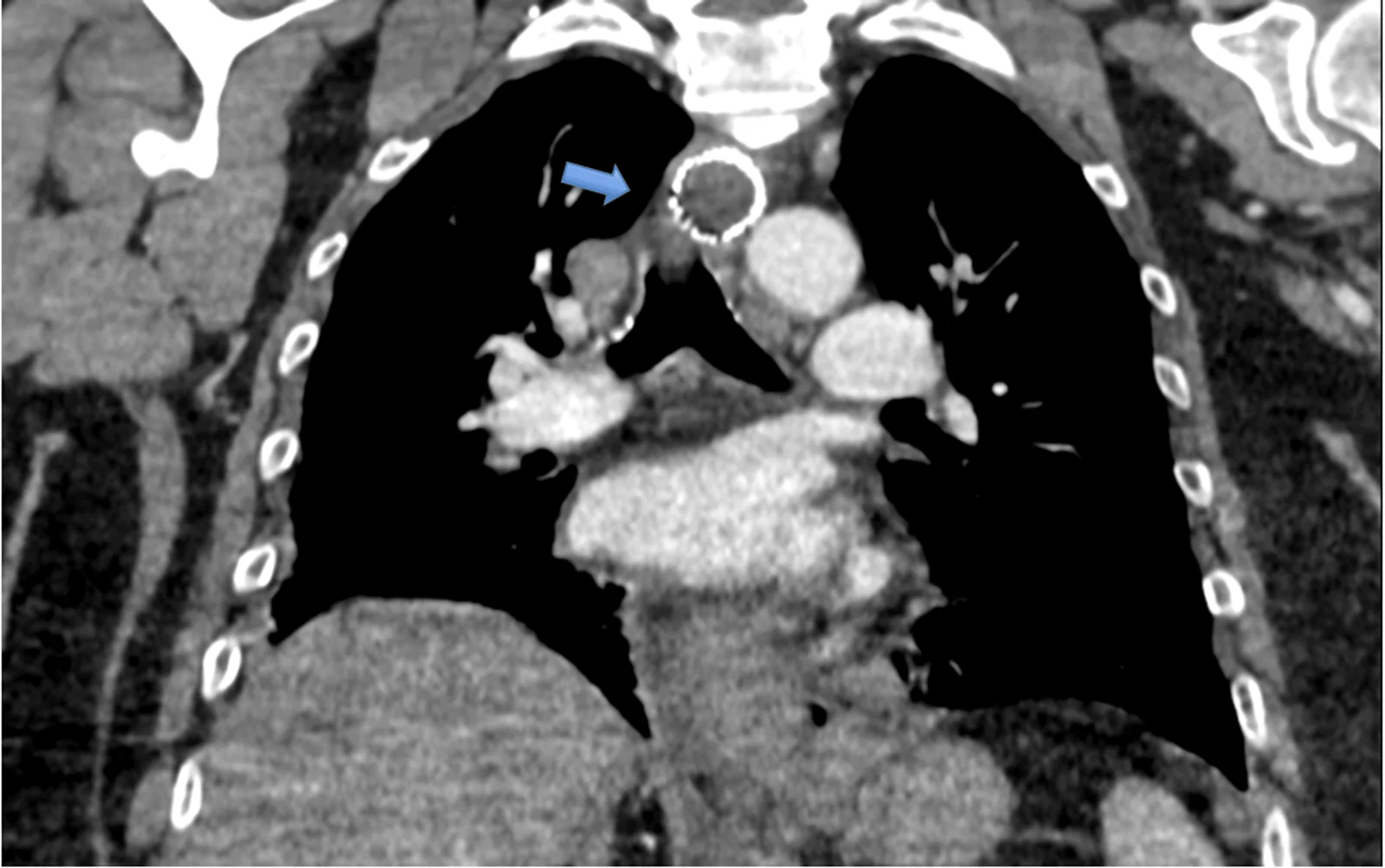
Computed tomography demonstrating proximal migration of the esophageal stent. Coronal view showing the migrated stent extending toward the hypopharynx and upper esophagus (arrow).

## Discussion

Esophageal perforation is a rare and possibly life-threatening event with approximately a 20% survival rate [7]. It may be caused by trauma, tumors, iatrogenic causes, spontaneous events, or foreign bodies [1]. Ingested foreign bodies are responsible for 9-35% of all esophageal perforations [8]. The diagnosis usually requires radiologic and endoscopic examination. After the diagnosis, the viable treatments for extracting the foreign body and repairing the perforation are several. The appropriate treatment may be endoscopic, surgical, or combined, depending on the level of the perforation, the timing of the intervention after the perforation, the co-morbidities of the patient, and the available resources. Self-expandable metal stents can be a useful treatment choice for patients in these medical conditions; however, delayed complications can occur, including migration of the stent [9].

In the multicenter retrospective study by Thomas et al., which evaluated migration rates of fully covered esophageal stents in benign and malignant disease, stent migration was reported to be significantly more frequent in benign indications compared with malignant strictures. This finding was attributed to the absence of a fixed obstructive lesion, limited tissue ingrowth, and the intrinsic design of fully covered stents, which, although advantageous for removability and effective sealing of perforations, provide less anchorage to the esophageal wall [10]. 

Importantly, while distal migration is well documented and frequently observed in both benign and malignant settings, reports of true proximal migration of esophageal stents are exceedingly rare. To date, the literature is primarily limited to isolated case reports describing such upward displacements, with only a few case reports documented (Table 2) [9,11]. Several other reports have described proximal displacement of esophageal stents; however, these cases involved different mechanisms, including stent fracture with migration of a stent fragment, erosion or prolapse through a tracheoesophageal fistula, or migration of stent components into the airway. These cases were therefore not considered true proximal migration of an intact esophageal stent.

**Table 2:** Previously reported cases of true proximal migration of an esophageal stent. Abbreviations: SEMS, self-expandable metal stent; NR, not reported

Study	Patient	Underlying disease	Stent	Fixation	Possible trigger/mechanism	Airway obstruction	Clinical outcome
Condron et al., 2009	Middle-aged man	Esophageal stenosis	Esophageal stent; exact type not reported	Not reported	True proximal stent migration	No	Proximal migration complicated by thoracic spondylodiscitis; the migrated stent was subsequently managed as a foreign body.
Sloan et al., 2014	72-year-old man	Cervical esophageal stricture following extensive oropharyngeal surgery, chemotherapy and radiotherapy for tongue cancer	Covered, retrievable polyurethane SEMS with flared ends	Not reported	True proximal migration into the hypopharynx; worsening cough during the preceding week was considered a possible contributing factor	Yes – severe, life-threatening upper-airway obstruction with glottic obstruction, hypoxia, and stridor	Emergency retrieval using video laryngoscopy and Magill forceps, with immediate resolution of respiratory distress. No further respiratory complications; the stent was not replaced, and gastrostomy was performed.

Several mechanical and anatomical factors may contribute to proximal stent migration. In addition to the increased retrograde intraluminal pressure generated during vomiting or retching, the effectiveness of the fixation method is likely to play an important role. Through-the-scope (TTS) clips may provide limited anchoring strength and less secure tissue capture than endoscopic suturing or over-the-scope clips, as their tissue-grasping depth and resistance to traction are relatively limited. These considerations may be particularly relevant when stents are deployed across benign perforation sites without an underlying stricture, where the absence of a fixed stenotic segment provides less mechanical resistance to stent displacement. Repetitive episodes of vomiting and coughing may further increase the mechanical forces acting on the stent and consequently facilitate proximal migration. From a preventive standpoint, appropriate stent fixation, together with the prevention and prompt treatment of vomiting and excessive coughing, may help reduce the risk of migration. The use of appropriate antiemetic therapy and careful management of cough should therefore be considered in patients at increased risk.

However, there is still a significant gap in the understanding of this uncommon but clinically relevant complication, which supports the need for heightened clinical vigilance and further research into both its mechanisms and prevention.

## Conclusions

Esophageal stents may provide an important therapeutic tool for cases with esophageal perforation. While most cases of stent migration involve movement in the distal direction, they may also proximally migrate causing life- threatening events such as acute airway obstruction. This case highlights the importance of appropriate stent fixation, close clinical monitoring, and appropriate perioperative management. Rapid endoscopic retrieval is essential when proximal migration occurs, allowing timely resolution of this potentially life-threatening complication.
